# Cancer treatment and the risk of cancer death among Aboriginal and non-Aboriginal South Australians: analysis of a matched cohort study

**DOI:** 10.1186/s12913-019-4534-y

**Published:** 2019-10-29

**Authors:** David Banham, David Roder, Marion Eckert, Natasha J. Howard, Karla Canuto, Alex Brown, for the CanDAD Aboriginal Community Reference Group and other CanDAD Investigators

**Affiliations:** 1 Wardliparingga Aboriginal Research Unit, South Australian Health and Medical Research Institute, Adelaide, Australia; 2 School of Health Sciences, Cancer Research Institute, University of South Australia, Adelaide, Australia; 3 Rosemary Bryant AO Research Centre, School of Nursing and Midwifery, University of South Australia North Terrace, Adelaide, Australia; 4 Aboriginal Health Research Group, Cancer Research Institute, University of South Australia, Adelaide, Australia

**Keywords:** Cancer treatment, Aboriginal, Indigenous, Cancer, Disparity, Survival

## Abstract

**Background:**

Aboriginal and Torres Strait Islander Australians have poorer cancer outcomes than other Australians. Comparatively little is known of the type and amount of cancer treatment provided to Aboriginal and Torres Strait Islander people and the consequences for cancer survival. This study quantifies the influence of surgical, systemic and radiotherapy treatment on risk of cancer death among matched cohorts of cancer cases and, the comparative exposure of cohorts to these treatments.

**Methods:**

Cancers registered among Aboriginal South Australians in 1990–2010 (*N* = 777) were matched with randomly selected non-Indigenous cases by sex, birth and diagnostic year, and primary site, then linked to administrative cancer treatment for the period from 2 months before to 13 months after diagnosis. Competing risk regression summarised associations of Indigenous status, geographic remoteness, comorbidities, cancer stage and treatment exposure with risk of cancer death.

**Results:**

Fewer Aboriginal cases had localised disease at diagnosis (37.2% versus 50.2%) and they were less likely to: experience hospitalisation with cancer diagnosis, unadjusted odds ratio (UOR) = 0.76; 95%CI = 0.59–0.98; have surgery UOR = 0.65; 95%CI = 0.53–0.80; systemic therapies UOR = 0.64; 95%CI = 0.52–0.78; or radiotherapy, UOR = 0.76; 95%CI = 0.63–0.94. Localised disease carried lower risk of cancer death compared to advanced cases receiving surgery or systemic therapies, SHR = 0.34; 95%CI = 0.25–0.47 and SHR = 0.35; 95%CI = 0.25–0.48. Advanced disease and no treatment carried higher risk of cancer death, SHR = 1.82; 95%CI = 1.26–2.63.

**Conclusion:**

The effects of treatment did not differ between Aboriginal and non-Indigenous cohorts. However, comparatively less exposure to surgical and systemic treatments among Aboriginal cancer cases further complicated the disadvantages associated with geographic remoteness, advanced stage of disease and co-morbid conditions at diagnosis and add to disparities in cancer death. System level responses to improving access, utilisation and quality of effective treatments are needed to improve survival after cancer diagnosis.

## Background

Australia’s Aboriginal and Torres Strait Islander population experience higher burden of disease from cancer compared to other Australians [1–3]. This excess burden is a function of diagnosis at younger average ages but lower cancer survival rates [4–7]. The disparities in survival are significantly influenced by widely reported factors such as comorbid conditions which pre-exist cancer diagnosis [8–10] and cancers often being diagnosed at relatively advanced stages with the cancer having spread to other tissue or organs [1, 2, 11]. Other disparities occurring after cancer diagnosis also exist. Ethnic disparities in the treatment of cancer are also implicated and have been the subject of international research and analysis for decades in the US [12] and other developed countries [13] for a wide range of primary cancer sites [14–21] and treatment modalities including surgery [14, 15], systemic therapies [16, 18], and radiotherapy [16, 18]. Studies in New Zealand found that Indigenous people (Maori) received less surgery for colon [22], breast [23, 24] and prostate [25] cancers, which increased their risk of cancer death and even accounted for survival disparities in some analyses [22, 24].

Australia’s first review of cancer treatments among Aboriginal and Torres Strait Islander people reported that poor survival outcomes were accompanied by lower rates of cancer hospitalisation [4]. Subsequent analyses within Australian states and territories, focussing on lung cancer and using person-linked cancer registry and hospital records, found surgery was clearly less frequent among Aboriginal and Torres Strait Islander people in Western Australia (WA) [26, 27], New South Wales (NSW) [28], the Northern Territory (NT) [29] and Queensland [30]. In Queensland, Aboriginal and Torres Strait Islander people with head and neck cancers were less like to receive surgery [31], and those with cervical cancer were less likely to receive optimal treatment [30] than other Australians. Exposure to breast cancer surgery was more equivocal with no differences reported in Queensland [32], but with lower odds of surgery reported among Aboriginal women in NSW [33]. Two matched cohort studies in Queensland considered cancer treatment more generally [8, 9] and found Aboriginal and Torres Strait Islander patients were less likely to receive surgery, chemotherapy and radiotherapy compared to cases among other Australians for the same primary sites, similar ages, and diagnostic periods of time. The observed lesser treatment of cancer was considered to pose a significant risk to cancer survival in each study [8, 9, 28, 29, 33].

While hospitals continue to provide most surgical treatment for cancer, the delivery of some other treatments has changed over time and moved from requiring hospital admission to outpatient and community settings. To obtain a comprehensive picture, hospital data can be linked with medical claims data, such as those from the Australian Pharmaceutical Benefits Scheme (PBS) and Medicare Benefits Schedule (MBS) [34].

To confirm the continued existence of disparities in cancer outcomes for Aboriginal and Torres Strait Islander people in South Australia (SA), to better determine the reasons for disparities and to inform changes in cancer care, an extended data system was developed by linking cancer registry, hospital, PBS and MBS treatment records in a Cancer Data and Aboriginal Disparities (CanDAD) project [35]. CanDAD aimed to develop and test an integrated cancer monitoring and surveillance system by incorporating epidemiological and narrative data to advocate and support health system change [35, 36]. To date CanDAD has described the associations between Indigenous status, geographic remoteness, cancer stage at diagnosis and comorbid conditions with cancer survival [1, 10].

In this study, we extend those earlier analyses by including uptake of cancer treatments. Specifically, we quantify the effect of, and exposure to, three treatment modalities: surgery, systemic therapy and radiotherapy on cancer death among matched population-based cohorts of Aboriginal and non-Indigenous cancer cases in South Australia.

## Methods

### Study governance

Aboriginal health research in South Australia is guided by a principled approach to research conduct and governance by Aboriginal community representatives [37]. The CanDAD project’s governance included a study specific Aboriginal Community Reference Group [38].

### Study design, setting and participants

This study utilised a retrospective matched cohort design focused on cancer cases diagnosed within the South Australian population. Situated in southern, central Australia, South Australia comprises a land area of almost one million square kilometres and a resident population of 1.7 million, of whom 71% live in the capital’s metropolitan area [38]. The Aboriginal and Torres Strait Islander population makes up 2.3% of the population with one-half living in non-metropolitan areas [38]. All cancer cases diagnosed among Aboriginal and Torres Strait Islander people in South Australia during the period 1990 to 2010 were categorised as such. Of those 777 cases, none were described as Torres Strait Islanders, therefore this cohort is referred to as the Aboriginal cohort. Each case in the Aboriginal cohort was matched with a cancer case involving a non-Indigenous person on the basis of: gender; birth and diagnosis year [39], and primary cancer site.

### Data sources and measurements

Cancer data were obtained from the South Australian Cancer Registry (SACR), a population-based registry coding cancer diagnoses to the International Classification of Diseases for Oncology (ICD-O-3) [40] and deaths to ICD-10. A broad definition of cancer death [41] was adopted to avoid potential misattribution to other organ sites and undercounting of deaths [10, 42]. Cancer stage at diagnosis was summarised using SEER methods [43] as: *localised -* confined to tissue of origin; *regional -* invaded adjacent tissue or regional nodes; *distant -* spread to distant lymph nodes or other organs, or to leukaemia (C42.1); and *unknown* stage where insufficient staging data were available.

The identification of Aboriginal and Torres Strait Islander people in administrative health records relies on self-identification by the individual. As the propensity to self-report as Aboriginal or Torres Strait Islander can vary across settings and time [44], it can be a source of misclassification bias [45]. We optimised the specificity of Aboriginal and Torres Strait Islander status by erring towards non-Indigenous status when uncertainty existed after cross-referencing SACR records against other linked datasets in the study [39], and following additional hand searching [35]. Some misclassification of Indigenous status may have resulted as a consequence of this practice, but we believe the low proportion of non-Indigenous cases that would have been misclassified would have had little effect on comparisons by Indigenous status.

SACR records area of residence categorised as geographically remote and not-remote (Major Cities and Inner/Outer Regional areas) using the Accessibility/Remoteness Index of Australia [46]. Person-linked hospitalisations for cohort cases during the period 1 July 1991 to 30 June 2013; included International Classification of Diseases (ICD-10-AM) [47] coded diagnoses extracted from the Integrated SA Activity Collection (ISAAC) and Alice Springs Hospital in the NT [35]. Comorbid health conditions recorded to the time of cancer diagnosis were coded using the Elixhauser Comorbidity Index (ECI) [10, 48].

An Australian standard classification system [49] arranges hospital procedures into blocks by level of invasiveness from: examination; insertion and removal; incision; to destruction or excision. We categorised surgery as procedures of most destruction or excision occurring in hospitalisations 2 months before to 13 months after the SACR recorded month of cancer diagnosis. The second treatment mode was systemic cancer therapies covering antineoplastic and immune-modulating agents. These were determined using hospital records (diagnoses Z511/2 &/or procedure blocks 1920) and person-linked PBS records after 1 July 2002 (Anatomic Therapeutic Classification codes L01, L02 and L03). Radiotherapy notifications were obtained from: the SACR; hospitalisations (diagnosis Z510 &/or procedure blocks 1786 to 1799); and person-linked MBS records (using broad type of service category K). Further treatment categories were derived where cases received all three treatment modes, or none of the three treatments.

### Outcomes

Survival time was assessed from cancer diagnosis to cancer death, or the close of follow-up at 31 December 2011.

### Statistical analysis

Analyses were performed using Stata 14 [50] within the Secure Unified Research Environment [51]. Bivariate associations between Indigenous status, demographic and cancer related variables were examined using cross-tabulations and conditional logistic regression. The latter regression technique was specifically developed to account for matched designs in analyses focused on binary outcomes (for example, yes/no to living in remote areas, or yes/no to receiving surgery) [52, 53]. We used it to derive the unadjusted odds ratios for Aboriginal rather than non-Indigenous cohort cases being included in a particular category of socio-demographic and clinical variables. Associations within each treatment mode were also cross-tabulated and the *p*-values of Pearson’s Chi-Square Test reported. The adjusted odds of receiving treatment modalities were concurrently assessed by Indigenous status and localised/non-localised stage at diagnosis using conditional logistic regression analyses to appropriately account for the matched design.

Multivariable analysis of the risk of cancer death is reported using sub-hazard ratio (SHR) estimates. We included all cohort cases in each model and adjusted for the competing risk of non-cancer mortality in a manner consistent with Fine and Gray’s approach [54] by using Stata’s *stcrprep* with *stcox*. Baseline Model 1 was adapted from our previously published regression models [10] with interaction terms for Indigenous status as exposure variable and geographic remoteness as covariate, together with comorbidity (Elixhauser) category and stage at diagnosis as moderators. Having found interactions between stage at diagnosis and treatment effects in each treatment modality, we categorised all cohort cases into one of three mutually exclusive groups for reporting and easy interpretation. These categories were: local stage at diagnosis with/without the relevant treatment modality; not local stage but receiving treatment; and, not local stage but not receiving treatment. Model 2 reports stage at diagnosis with and without surgical treatment. Models 3 (systemic therapy); 4 (radiotherapy); 5 (cases receiving all three treatment modes); and 6 (none of the three treatment modes) were similarly reported. Model 7 regrouped treatment/stage at diagnosis groupings and further considered the combined effects of surgery, systemic and radiotherapies by stage at diagnosis. Each model’s parsimony and fit to the cohort data were considered using Bayesian Information Criterion (BIC) statistics [55]. The sensitivity of results were assessed by confining cases to those with hospitalisations with principal diagnoses of cancer. All Models were assessed to ensure adherence to the proportional hazards assumption using Schoenfeld residuals [56].

## Results

**Fig. 1:**
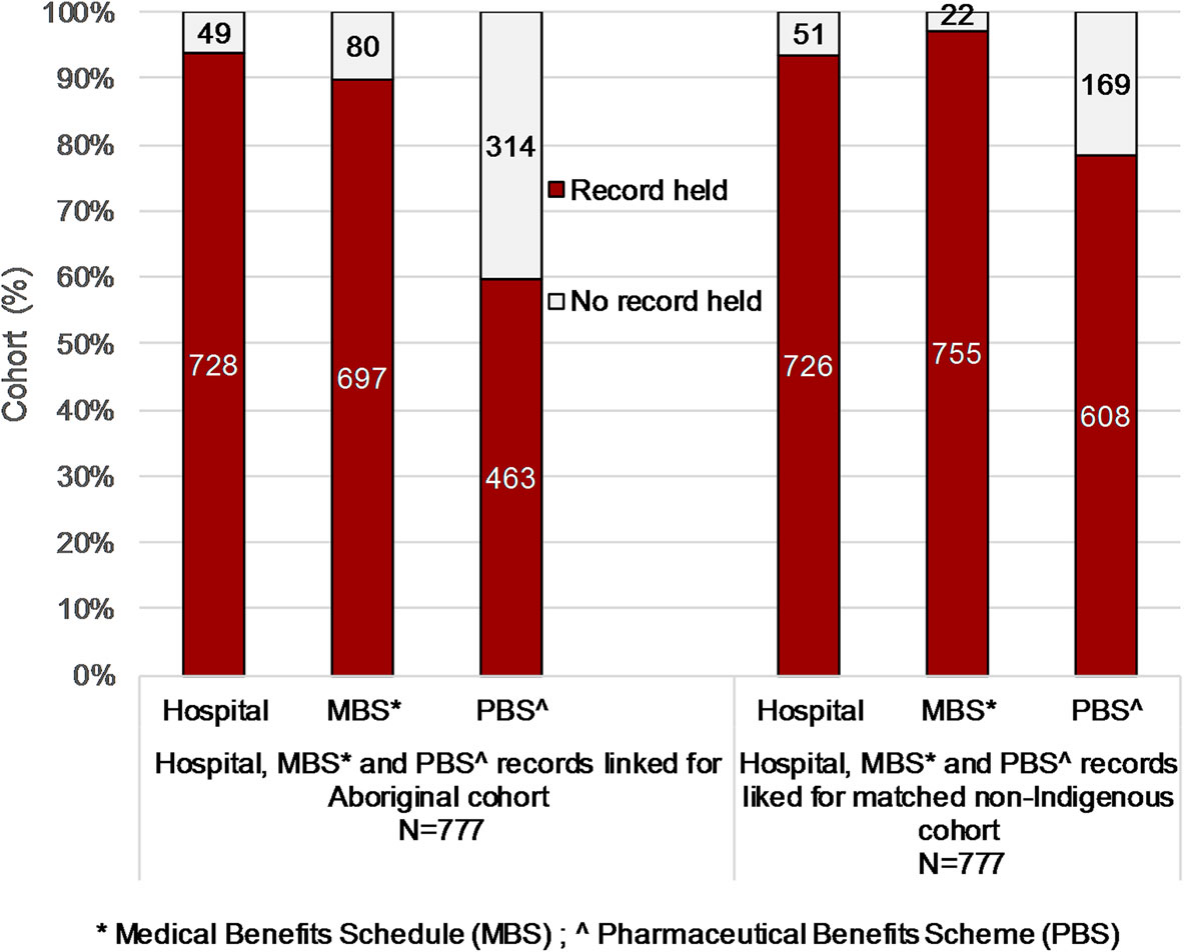
Linkage of hospital, medical benefits and pharmaceutical benefits

The Aboriginal (*N* = 777) and matched non-Indigenous cancer cohorts were equivalent by matching variables (that is: year of birth; sex; year of diagnosis; and, primary cancer site). Hospital, MBS and PBS records were linked for 94.6, 93.4 and 68.9% of cohort cases respectively. Aboriginal cases were less often linked to MBS and PBS records than matched non-Indigenous cases, χ^2^(2) = 35.3 *p* < 0.001 and χ^2^(2) = 63.2 p < 0.001 respectively (Fig. 1). However, linkage of records of Aboriginal cohort cases improved over time for example, from 81% for PBS records in the period 2002 to 2006 to 90% from 2007.

The contemporary population setting of cancer in SA [1] and cohort differences are more fully detailed elsewhere [1, 10] but in summary, Aboriginal cases were more likely to live in geographically remote areas, have more comorbid conditions and more advanced cancer stage at diagnosis (Table 1). Aboriginal cases also had less likelihood of hospitalisation with a principal diagnosis of cancer in the treatment period, OR (unadjusted) = 0.76; 95%CI = 0.59–0.98 and consistently lower odds of receiving surgery, systemic therapy or radiotherapy, or all three treatment modalities. Conversely, Aboriginal cases were more likely to have *none* of the three treatment modes recorded, OR (unadjusted) = 1.87; 95%CI = 1.44–2.43.

**Table 1:** Modes of cancer treatment by Aboriginality, demographics and clinical characteristics

		Aboriginal cohort		Matched non-Indigenous cohort			
		N	%			Odds Ratio (unadjusted)	95%CI
Total		777	100.0%	777	100.0%		
Matching variables							
Age							
<50 years		212	27.3%	190	24.5%		
50-69 years		400	51.5%	408	52.5%		
70+ years		165	21.2%	179	23.0%		
Sex							
Male		375	48.3%	375	48.3%		
Female		402	51.7%	402	51.7%		
Primary site							
Colorectal (C18-C21)		77	9.9%	77	9.9%		
Lung (C33-C34)		106	13.6%	106	13.6%		
Breast (C50 & Female)		77	9.9%	77	9.9%		
Cervix (C53)		24	3.1%	24	3.1%		
All other sites		493	63.4%	493	63.4%		
Socio-demographic and clinical variables							
Geographically remote							
No		606	78.0%	742	95.5%	1.00	Reference
Yes		171	22.0%	35	4.5%	5.98	4.09-8.74
Comorbid conditions (Elixhauser)							
0-3		667	85.8%	742	95.5%	1.00	Reference
4+		110	14.2%	35	4.5%	3.50	2.35-5.19
Summary stage at diagnosis							
Localised		289	37.2%	390	50.2%	1.00	Reference
Regional		155	19.9%	132	17.0%	1.58	1.20-2.09
Distant/Unstageable		333	42.9%	255	32.8%	1.76	1.41-2.20
Treatment modality							
Hospitalisation with primary diagnosis of cancer	Received	617	79.4%	649	83.5%	0.76	0.59-0.98
	Not received	160	20.6%	128	16.5%	1.00	Reference
Surgery	Received	408	52.5%	488	62.8%	0.65	0.53-0.80
	Not received	369	47.5%	289	37.2%	1.00	Reference
Systemic therapy	Received	252	32.4%	334	43.0%	0.64	0.52-0.78
	Not received	525	67.6%	443	57.0%	1.00	Reference
Radiotherapy	Received	309	39.8%	360	46.3%	0.76	0.63-0.94
	Not received	468	60.2%	417	53.7%	1.00	Reference
Each of surgery, systemic and radiotherapy	Received	77	9.9%	140	18.0%	0.50	0.37-0.67
	Not received	700	90.1%	637	82.0%	1.00	Reference
No surgery, systemic or radiotherapy	Received	183	23.6%	110	14.2%	1.87	1.44-2.43
	Not received	594	76.4%	667	85.8%	1.00	Reference
Vital status^b^							
Alive		220	28.3%	349	44.9%	1.00	Reference
Cancer death		461	59.3%	340	43.8%	2.15	1.73-2.68
Non-cancer death		96	12.4%	88	11.3%	1.73	1.24-2.42

^a^up to 2 months before and 13 months after month of diagnosis

^b^to censoring of observations at 31 December 2011

Table 2 further describes the distribution of person, area of residence and clinical characteristics receiving each treatment mode within each cohort. In both cohorts, older age was associated with relatively fewer cases receiving all three treatments (that is surgery and systemic and radiotherapy) while it was reciprocally associated with increased numbers receiving none of the three treatments. Cases not having surgery or radiotherapies were relatively more common when disease stage at diagnosis was distant, unstageable or undefined. Other statistically significant differences within the Aboriginal cohort compared to non-Aboriginal included less surgery among older cases, more reports of radiotherapy among distant/unstageable cases and increased reports of no treatment among Aboriginal males and those living in areas of most disadvantage or geographic remoteness. Female breast cancers in both cohorts were relatively more likely to receive surgery and systemic and radiotherapy.

**Table 2:** Treatment modality received by Aboriginality, demographic and clinical variables

	Hospitalised with primary diagnosis of cancer			Received surgery			Received systemic therapy			Received radiotherapy			Received each of surgery, systemic and radiotherapy			Reveived no surgery, systemic or radiotherapy		
	Aboriginal cohort	Matched non-Indigenous cohort	p	Aboriginal cohort	Matched non-Indigenous cohort	p	Aboriginal cohort	Matched non-Indigenous cohort	p	Aboriginal cohort	Matched non-Indigenous cohort	p	Aboriginal cohort	Matched non-Indigenous cohort	p	Aboriginal cohort	Matched non-Indigenous cohort	p
Total																		
n	617	649		408	488		252	334		309	360		77	140		183	110	
column %	100.0%	100.0%		100.0%	100.0%		100.0%	100.0%		100.0%	100.0%		100.0%	100.0%		100.0%	100.0%	
Age																		
<50 years	28.5%	25.0%	0.17	32.8%	26.0%	0.07	31.0%	27.2%	0.96	29.4%	25.6%	0.50	35.1%	26.4%	0.38	19.7%	18.2%	0.23
50-69 years	53.2%	53.2%		52.2%	55.9%		53.6%	58.7%		54.0%	56.1%		54.5%	60.0%		46.4%	38.2%	
70+ years	18.3%	21.9%		15.0%	18.0%		15.5%	14.1%		16.5%	18.3%		10.4%	13.6%		33.9%	43.6%	
Sex																		
Male	47.2%	48.1%	0.72	42.6%	45.7%	0.36	44.8%	48.5%	0.38	52.1%	49.7%	0.54	45.5%	42.1%	0.64	54.6%	48.2%	0.28
Female	52.8%	51.9%		57.4%	54.3%		55.2%	51.5%		47.9%	50.3%		54.5%	57.9%		45.4%	51.8%	
Primary site																		
Colorectal (C18-C21)	11.2%	11.1%	0.92	13.2%	13.3%	0.55	12.7%	10.8%	0.86	7.4%	6.4%	0.66	15.6%	8.6%	0.31	8.2%	6.4%	0.39
Lung (C33-C34)	13.9%	13.9%		5.4%	7.8%		12.7%	12.6%		19.1%	20.3%		3.9%	10.0%		14.2%	12.7%	
Breast (C50 & Female)	10.0%	10.9%		13.7%	13.9%		18.3%	17.4%		13.3%	14.2%		32.5%	30.0%		4.9%	3.6%	
Cervix (C53)	3.1%	2.3%		4.2%	2.9%		1.2%	2.1%		4.9%	2.8%		1.3%	1.4%		1.1%	4.5%	
All others	61.8%	61.8%		63.5%	62.1%		55.2%	57.2%		55.3%	56.4%		46.8%	50.0%		71.6%	72.7%	
Geographically remote																		
No	76.7%	95.5%	<0.01	78.4%	95.7%	<0.01	86.1%	94.3%	<0.01	80.9%	94.4%	<0.01	80.5%	95.7%	<0.01	68.9%	98.2%	<0.01
Yes	23.3%	4.5%		21.6%	4.3%		13.9%	5.7%		19.1%	5.6%		19.5%	4.3%		31.1%	1.8%	
Comorbid conditions (Elixhauser)																		
0-3	84.3%	95.2%	<0.01	85.8%	96.9%	<0.01	88.9%	96.4%	<0.01	88.7%	96.1%	<0.01	94.8%	97.1%	0.38	86.3%	91.8%	
4+	15.7%	4.8%		14.2%	3.1%		11.1%	3.6%		11.3%	3.9%		5.2%	2.9%		13.7%	8.2%	
Summary stage at diagnosis																		
Localised	34.2%	49.8%	<0.01	40.0%	55.7%	<0.01	32.9%	43.1%	0.04	40.5%	46.7%	0.25	36.4%	45.7%	0.39	29.0%	46.4%	0.01
Regional	21.9%	18.6%		26.0%	20.1%		24.2%	20.7%		24.3%	22.8%		29.9%	27.1%		12.0%	9.1%	
Distant/Unstageable	43.9%	31.6%		34.1%	24.2%		42.9%	36.2%		35.3%	30.6%		33.8%	27.1%		59.0%	44.5%	
Vital status^b^																		
Alive	29.0%	45.6%	<0.01	38.0%	55.9%	<0.01	35.7%	44.9%	0.08	29.4%	41.1%	<0.01	40.3%	48.6%	0.50	12.0%	26.4%	<0.01
Cancer death	60.0%	45.0%		48.3%	35.2%		57.5%	49.4%		62.1%	52.5%		55.8%	47.9%		73.8%	48.2%	
Non-cancer death	11.0%	9.4%		13.7%	8.8%		6.7%	5.7%		8.4%	6.4%		3.9%	3.6%		14.2%	25.5%	

^a^up to 2 months before and 13 months after month of diagnosis

^b^to censoring of observations at 31 December 2011

Totals may not always sum to 100 % because of rounding

The relationships between receiving treatment and the concurrent effects of stage at diagnosis and Indigenous status are summarised in Table 3. Localised/not localised stage at diagnosis did not predict receipt of any of the treatment modes. However, being Aboriginal was associated with lower likelihood of receiving each treatment mode. Indeed, Aboriginal cases had twice the odds of receiving none of the three treatment types compared to their non-Indigenous contemporaries, OR = 2.09; 95%CI = 1.55–2.81 adjusted for stage at diagnosis.

**Table 3:** Conditional logistic regression models relating receipt of each treatment mode to Indigenous status and localised stage at diagnosis

	Hospitalised with principal cancer diagnosis			Received surgery			Received systemic therapy			Received radiotherapy			Received surgery and systemic therapy and radiotherapy			Received no surgery or systemic or radiotherapy		
	Odds Ratios (adjusted^a^)	95% CI	*p* > |z|	Odds Ratios (adjusted^a^)	95% CI	*p* > |z|	Odds Ratios (adjusted^a^)	95% CI	*p* > |z|	Odds Ratios (adjusted^a^)	95% CI	*p* > |z|	Odds Ratios (adjusted^a^)	95% CI	*p* > |z|	Odds Ratios (adjusted^a^)	95% CI	*p* > |z|
Aboriginal^b^																		
Non-Indigenous	1.00	Ref		1.00	Ref		1.00	Ref		1.00	Ref		1.00	Ref		1.00	Ref	
Aboriginal	0.70	0.52–0.94	0.02	0.57	0.45–0.73	<0.001	0.53	0.42–0.68	<0.001	0.70	0.56–0.89	< 0.001	0.41	0.29–0.59	< 0.001	2.09	1.55–2.81	<0.001
Stage at diagnosis																		
Not localised	1.00	Ref		1.00	Ref		1.00	Ref		1.00	Ref		1.00	Ref		1.00	Ref	
Localised	0.72	0.44–1.17	0.19	1.05	0.72–1.55	0.79	0.74	0.50–1.10	0.14	0.90	0.63–1.30	0.59	0.76	0.44–1.32	0.33	0.85	0.52–1.38	0.51

^a^Concurrent adjustment for Indigenous status and stage at diagnosis

^b^Main effect of Indigenous status the sole, significant predictor of treatment receipt in these models

Table 4 summarises the risk of cancer death using sub-hazard ratios (SHR). Using cases among Aboriginal people in non-remote settings as the reference group, Baseline Model 1 indicates those cases experienced a lower risk of cancer death than Aboriginal cases from geographically remote areas, but significantly greater risk than non-Indigenous cases from non-remote areas. Cancer stage was highly predictive of the risk of cancer death, as was the presence of 4 or more comorbid conditions [10].

**Table 4:** Competing risk regression analyses for cancer survival among Aboriginal and matched non- Indigenous cohorts by discrete treatment type, Models 1 to 4

	Model 1 - baseline^a^			Model 2 - surgery among cohorts			Model 3 - systemic therapy among cohorts			Model 4 - radiotherapy among cohorts		
	Subhazard Risk Ratio	95% CI	*p* > |z|	Subhazard Risk Ratio	95% CI	*p* > |z|	Subhazard Risk Ratio	95% CI	*p* > |z|	Subhazard Risk Ratio	95% CI	*p* > |z|
Aboriginal x geographic remoteness non- Indigenous												
not Remote	0.67	0.56–0.80	<0.001	0.70	0.59–0.83	<0.001	0.68	0.57–0.81	<0.001	0.68	0.57–0.80	<0.001
Remote	0.87	0.40–1.87	0.71	0.90	0.39–2.07	0.81	0.92	0.42–1.98	0.82	0.90	0.41–2.01	0.80
Aboriginal												
not Remote	1.00	Reference		1.00	Reference		1.00	Reference		1.00	Reference	
Remote	1.65	1.13–2.41	<0.01	1.70	1.16–2.49	<0.01	1.62	1.12–2.35	0.011	1.76	1.22–2.54	<0.01
Comorbidity score (Elixhauser)												
0–3	1.00	Reference		1.00	Reference		1.00	Reference		1.00	Reference	
4+	1.58	1.08–2.31	0.02	1.65	1.13–2.42	0.01	1.49	1.01–2.21	0.05	1.53	1.04–2.24	0.03
Stage at diagnosis												
Local	1.00	Reference										
Regional	2.57	1.86–3.56	<0.001									
Distant	6.20	4.30–8.93	<0.001									
Unknown	4.61	2.66–8.00	<0.001									
Stage x treatment modality												
Local stage with/without treatment modality				0.34	0.25–0.47	<0.001	0.35	0.25–0.48	<0.001	0.26	0.19–0.36	<0.001
Not local stage with treatment modality				1.00	Reference		1.00	Reference		1.00	Reference	
Not local stage without treatment modality				2.05	1.48–2.85	<0.001	1.65	1.24–2.19	<0.01	1.07	0.79–1.43	0.667
BIC	641.4			635.9			641.0			648.0		

^a^Abridged Model 2 from Banham, et al. (2018) [10]

With minor variations in SHRs, Indigenous status by remoteness and 4 or more comorbidities contributed in a similar manner to each of the subsequent models derived. Stage at diagnosis also contributed a main effect within each model. We found no further interactions between Indigenous status and stage at diagnosis or treatment mode on the risk of cancer death.

However, interactions were found between stage at diagnosis and the relevant treatment mode. For example, in Model 2 cases with localised disease at diagnosis had significantly lower risk of cancer death than those in the reference category with more advanced stage of disease and who also received surgery, SHR = 0.34; 95%CI = 0.25–0.47. In turn, cases who had more advanced disease but did not receive surgery experienced twice the risk of cancer death, SHR = 2.05; 95%CI = 1.48–2.85 compared to those in the reference category. Similar effects were observed in Model 3 (systemic therapy) with lower risk of cancer death among cases with localised disease compared to the reference group with advanced stage disease and systemic treatment, SHR = 0.35; 95%CI = 0.25–0.48. Those with more advanced disease cancer but did not receive systemic therapy had higher risk of cancer death, SHR = 1.65; 95%CI 1.25–2.19. Model 4 (radiotherapy) also showed those with localised disease had lower risk of cancer death than those with more advanced disease and radiotherapy treatment, SHR = 0.26; 95%CI 0.19–0.36. Among those with more advanced disease, the risk of cancer death did not vary on the basis of receiving radiotherapy.

Table 5 includes Model 5 (receipt of surgery *and* systemic *and* radiotherapy). Again, localised disease at diagnosis was associated with lower risk of cancer death compared to those with more advanced disease who received all three treatment modalities, SHR = 0.38; 95%CI 0.24–0.58. In turn, others with advanced disease but who did not receive all three forms of treatment had higher average risk of cancer death, SHR = 1.62; 95%CI 1.09–2.41. Model 6 focusses on the opposite scenario where cases did not receive surgery, or systemic, or radiotherapies. In this case, localised disease was associated with lower risk when compared to the reference group of cases with more advanced disease and who received any of the three treatment modalities, SHR = 0.28; 95%CI 0.21–0.38. Compared to the same reference group, those with more advanced disease who did not receive any of the treatment modalities experienced significantly higher risk of cancer death. SHR = 1.82; 95%CI 1.26–2.63.

**Table 5:** Competing risk regression analyses for cancer survival among Aboriginal and matched non- Indigenous cohorts by discrete treatment type, Models 5 and 6

	Model 5 - surgery and systemic and radiotherapies among cohorts			Model 6 - no surgery or systemic or radiotherapies among cohorts		
	Subhazard Risk Ratio	95% CI	*p* > |z|	Subhazard Risk Ratio	95% CI	*p* > |z|
Aboriginal x geographic remoteness non-Indigenous						
not Remote	0.69	0.58–0.82	<0.001	0.70	0.59–0.83	<0.001
Remote	0.87	0.39–1.93	0.73	0.93	0.42–2.08	0.86
Aboriginal						
not Remote	1.00	Reference		1.00	Reference	
Remote	1.75	1.21–2.54	<0.01	1.64	1.12–2.40	<0.01
Comorbidity score (Elixhauser)						
0–3	1.00	Reference		1.00	Reference	
4+	1.53	1.04–2.24	0.03	1.57	1.07–2.32	0.02
Stage x treatment modality						
Local stage with/without treatment modality	0.38	0.24–0.58	<0.001			
Not local stage with treatment modality	1.00	Reference				
Not local stage without treatment modality	1.62	1.09–2.41	0.02			
Stage x treatment modality						
Local stage with/without any of treatment modalities				0.28	0.21–0.38	<0.001
Not local stage receiving *none* of the treatment modalities				1.82	1.26–2.63	<0.01
Not local stage receiving *any* of the treatment modalities				1.00	Reference	
BIC	644.7			641.4		

Model 7 simultaneously assessed reports of any of the three treatment modalities among cases (Table 6). Over and above the effects of Aboriginality, geographic remoteness and the number of comorbid conditions, the average risk of cancer death varied significantly across groups on the basis of stage at diagnosis and treatment received. With regards to surgery, cases receiving surgery regardless of their stage at diagnosis had lower risk (SHR = 0.56; 95%CI = 0.36–0.86) compared to the reference group of local stage without surgery, SHR = 1, which was not statistically different to those with more advanced disease who also did not receive surgery (SHR = 1.12; 95%CI 0.66–1.89). With regards to systemic therapy, cases with localised disease regardless of therapy receipt had lower average risk (SHR = 0.50; 95%CI = 0.33–0.76) than the reference group with more advanced disease and who received systemic therapy (SHR = 1.00). Risk was comparatively higher when stage was not localised and systemic therapy was not received, SHR = 1.55; 95%CI = 1.15–2.08. With respect to radiotherapy, cases with localised disease and no radiotherapy had lower risk (SHR = 0.39; 95%CI = 0.22–0.67) compared to those with more advanced disease and no radiotherapy, SHR = 1.00, and those receiving radiotherapy regardless of stage of disease (SHR = 1.11; 95%CI 0.82–1.50).

**Table 6:** Competing risk regression analyses for cancer survival among Aboriginal and matched non- Indigenous cohorts by concurrent treatment type

	Model 7 - any treatment mode		
	Subhazard Risk Ratio	95% CI	*p* > |z|
Aboriginal x geographic remoteness non- Indigenous			
not Remote	0.70	0.58–0.83	<0.001
Remote	0.77	0.37–1.61	0.49
Aboriginal			
not Remote	1.00	Reference	
Remote	1.53	1.02–2.28	0.04
Comorbidity score (Elixhauser)			
0–3	1.00	Reference	
4+	1.72	1.16–2.54	<0.01
Stage x treatment modality			
Any stage with surgery	0.56	0.36–0.86	<0.01
Local stage without surgery	1.00	Reference	
Not local stage without surgery	1.12	0.66–1.89	0.68
Local stage with/without systemic therapy	0.50	0.33–0.76	<0.001
Not local stage with systemic therapy	1.00	Reference	
Not local stage without systemic therapy	1.55	1.15–2.08	0.004
Local stage without radiotherapy	0.39	0.22–0.67	<0.001
Not local stage without radiotherapy	1.00	Reference	
Any stage with radiotherapy	1.11	0.82–1.50	0.51
BIC	650.2		

The lowest BIC values were observed for Model 2 (surgery) and Model 3 (systemic therapies) which indicated these as the best fitting, most parsimonious models. After constraining cases to only include in-hospital principal diagnosis of cancer within the treatment period, receipt of surgery remained associated with lowest risk of cancer death, SHR = 0.31; 95%CI = 0.22–0.46. Stratification using broad primary site groupings were also tested with little change to Model 2 and 3 parameters were observed. The proportional hazard assumption was met in each of the reported Models 1 to 7.

**Fig. 2:**
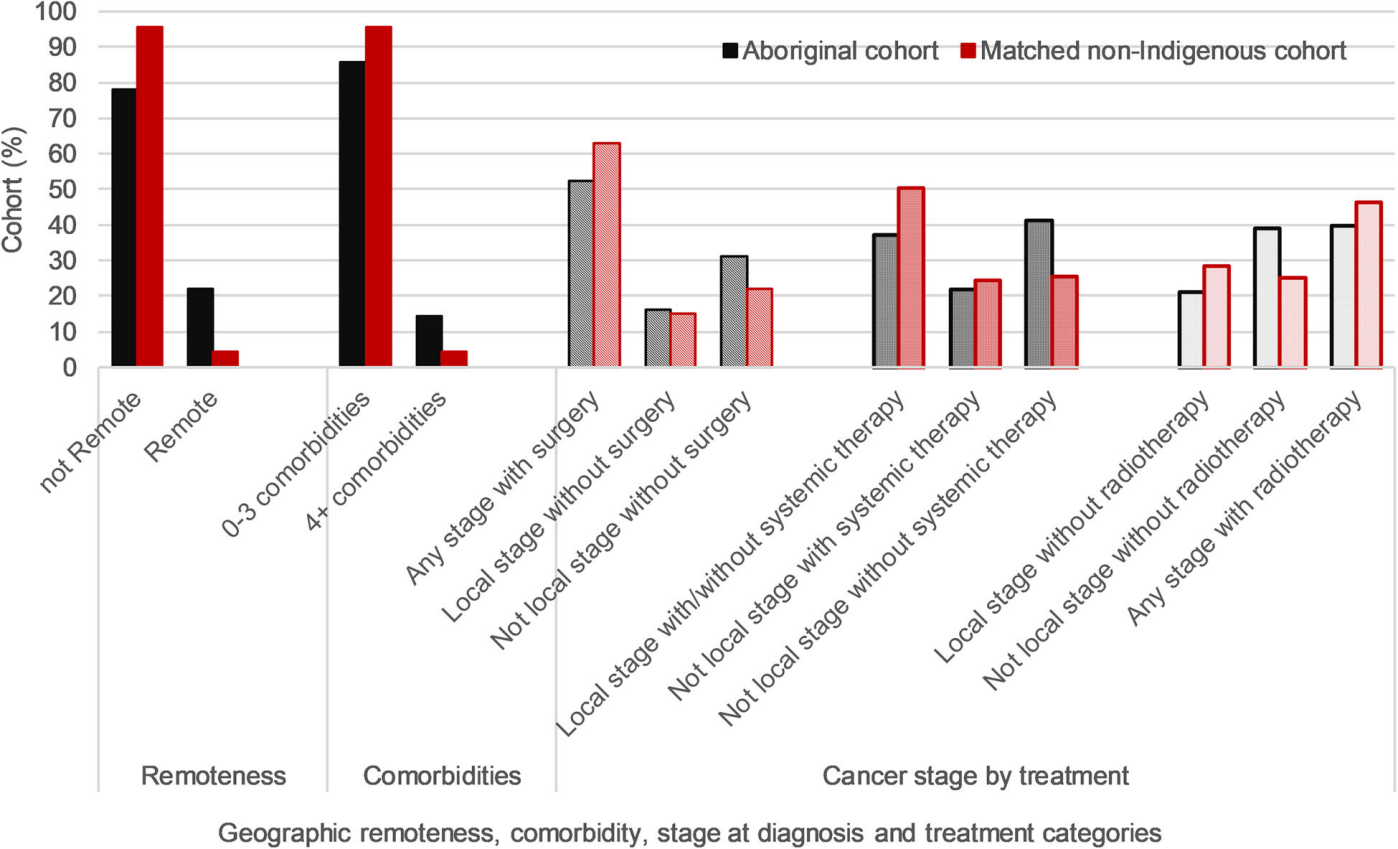
Exposure of risks associated with cancer death by cohort (Model 7)

Figure 2 summarises the exposure to stage and treatment categories associated with more and less risk of cancer death. Relatively more Aboriginal cases were exposed to higher risk of cancer death associated with living in geographically remote areas and having four or more comorbid conditions. Fewer Aboriginal cases were exposed to stage and treatment categories associated with less risk. For example, fewer Aboriginal cases than non-Indigenous received surgery (52.5% versus 62.8%). Conversely, Aboriginal cases with advanced stage tumours that did not receive surgery were comparatively more common (31.3% versus 22.0% of non-Indigenous cases) while 16% more Aboriginal cases had more advanced tumours but did not receive systemic therapies (41.1% versus 25.4%).

## Discussion

This population-based study of Aboriginal and non-Indigenous cancer cases (matched by sex, age, year of diagnosis and primary cancer site) affirmed that localised cancers were associated with lowest risk of cancer death. When cancers with more advanced stages were analysed, cases not receiving surgical treatment were found to have twice the risk of cancer death as cases having cancer surgery. Similarly, cases not receiving systemic therapies had a 65% higher risk of cancer death compared to those receiving these treatments. Radiotherapy appeared not to reduce the risk of death; however, where all three treatments were reported the risk of cancer death was reduced. Conversely, not receiving *any* of the three treatments was associated with an 82% greater risk of cancer death on average. No evidence was found of differential treatment effects for Aboriginal and non-Indigenous cases. What was clearly apparent though, was a widespread lower treatment of Aboriginal cases relative to their non-Indigenous matched contemporaries. Over and above the effects of stage at diagnosis and cancer treatment, being Aboriginal, living in remote locations and having multiple comorbid conditions remained associated with higher risk of cancer death.

The degree to which surgical and systemic treatments reduced the risk of cancer death in cases with more advanced disease is consistent with other Australian population studies. For example, receiving any cancer treatment reduced the risk of cancer death among head and neck cancer cases in Queensland by 20% [31] after adjusting for socio-demographic influences, stage, and comorbidities. In NSW, lung cancer surgery reduced risk of cancer death by around three-quarters, but Aboriginal and Torres Strait Island people faced 30% higher residual risk (SHR = 1.3) [28]. Also, the NT reported hazard ratios in the order of 1.5 [29] among Aboriginal and Torres Strait Islander cases with the most commonly diagnosed cancers after allowing for the effects of treatment and this is equivalent to the sub-hazard risk of 1.6 observed in our study. For cancers overall, elevated risk of cancer death among Aboriginal and Torres Strait Islander cases persisted in a matched Queensland cohort after allowance for area of residence, comorbidities, stage and treatment (HR = 1.3) and occasions where no surgical treatment carried additional risk (HR = 1.9) [8]. Our results differed in that reports of radiotherapy were associated with reduced risk of cancer death in Queensland (HR = 1.3 with no radiotherapy [8]) but this was not so in the SA cohort. This finding is not without precedent [17] and may be influenced by whether radiotherapy was intended to be curative, or palliative [57].

The lower exposure to the three treatment modes among Aboriginal compared to non-Indigenous cases in this study is consistent with the wider literature and poses a risk to cancer survival [29, 33]. The disparities ranged from lung cancers in WA where Aboriginal and Torres Strait Islander cases were 36% less likely than non-Indigenous cases to receive surgery [26, 27] to 14% lower treatment rates in Queensland [30] where the odds of receiving head and neck (20%) [31] and cervical cancer (19%) [30] treatments were also reported. Within similarly matched cohorts that considered all invasive cancers, surgery was reportedly 24% less likely among Aboriginal and Torres Strait Islander cases, as was chemotherapy (20%) and radiotherapy (9%). Similarly, we report greater odds (87%) of Aboriginal cases receiving no treatment compared to 38% in Queensland [9].

The differences in exposure to cancer treatment occurred in Australia, a setting where health care is available to all residents and the health system monitors performance to ensure equitable, timely access to services by all [58, 59]. Nonetheless, we found differences in access to healthcare exist. Firstly, Aboriginal cases up to 24% less likely to be hospitalised with a primary diagnosis of cancer than their non-Indigenous contemporaries. Secondly, once hospitalised, differences in utilisation and quality of care [60, 61] were apparent and fewer Aboriginal patients received surgery, systemic therapies or radiotherapy. Effective, systematic responses are needed to address these gaps in service access, utilisation and quality. Attending to patient experience [62] is one means of informing such responses while another is to promote improved communication, cultural competency, safety and collaboration among patients and clinicians more generally. A further avenue to informing responses would take advantage of electronic patient care records by flagging relevant system inputs affecting outputs and outcomes. For example, automatically flagging the need for an interpreter is a useful input to assist patient-centred exchanges with clinicians. Once treatment pathways are initiated, documenting the influences that affect uptake of those pathways, such as patient refusal and/or clinical contra-indications, will inform on what impediments to treatment uptake occur, where they occur in the pathway and who they involve.

While our analysis was strengthened by an efficient fixed effects design and allowance for competing risk from non-cancer death, low case numbers limited stratification by cancer sites. This highlights the desirability of pooling analyses across jurisdictions to maximise statistical precision and improve generalisability for a relatively small population with pressing needs. Nevertheless, the results are consistent with other results in the international and domestic literature using administrative hospital records. The study also enabled a piloting of broader data coverage by incorporating MBS and PBS with hospital admission data as potentially sustainable sources of information on cancer treatment in order to cover primary, community, acute and tertiary care settings).

Not only was the extended data system within CanDAD feasible, but it has enhanced our understanding of influences on the risk of cancer death among Aboriginal people in South Australia by supplementing existing information on likely effects of stage at diagnosis, geographic remoteness and comorbidity to include the effects of, and exposure to, cancer treatment types. Given the CanDAD project’s governance includes Aboriginal community representatives and South Australian Cancer Services, this information is directly available for system-wide cancer control [63] and chronic disease [64] initiatives benefiting Aboriginal and Torres Strait Islander people.

The results inform service planners on the lessor treatment of Aboriginal than non-Indigenous cases that heighten the risk of cancer death and negatively influence cancer survivals. This is important evidence for introducing corrective initiatives such as encouraging timely access and uptake of effective cancer care in accordance with national treatment guidelines [65] and maintaining monitoring activities within the health system to assess effectiveness. For example, it is feasible for CanDAD’s person-linked analyses to inform patient-centred enquiries and monitor exposure to treatment and associated outcomes of care. These data would complement those independently available on quality of care and productivity in the health system.

In using population cancer registry records as the basis for CanDAD’s data system, we matched primary site among Aboriginal and non-Indigenous cases on the basis of 3-digit ICD-O-3 coding. It is possible the reported differences in outcomes are influenced by different distributions of tumour subtypes within those sites. Given the non-Indigenous cases were drawn from a pool of 219,234 diagnoses [1], it is feasible to pursue tumour subtypes as an extra step in matching in future studies.

While the findings highlight the value of using available administrative records, two limitations with their use also emerged. Firstly, administrative practices saw less recording of systemic therapies within hospital funding collections from the 2007 financial year and an increased reliance on PBS records for enumerating these therapies. By 2007, Aboriginal cases were more consistently linked to PBS records which enabled continuity in assessing exposure to these therapies. A further limitation in evaluating exposure to treatments more generally is that PBS data do not inform on whether the reported treatment course was completed, nor could we assess time to treatment or partition treatments by curative intent. These issues raise further R&D opportunities to incorporate relevant clinical information into the CanDAD system with the aim of informing continuous quality improvement activities encouraging compliance with national guidelines and the uptake of optimal cancer care pathways [65].

## Conclusions

The fact that Aboriginal cancer patients were comparatively less likely to receive cancer treatment adds to disparities in cancer death and exacerbate the disadvantages that come from geographic remoteness, lack of cultural awareness, advanced stage of disease and multiple co-morbid conditions at diagnosis. A systemic response ensuring earlier access to cancer care facilities and maximising the utilisation and quality of effective cancer treatments should be actively pursued, to reduce the risk of cancer death and improve survival after cancer diagnosis for Aboriginal and Torres Strait Islander people.

## Data Availability

The datasets generated and/or analysed during the current study are not publicly available due to privacy reasons, including the provisions of the Australian Privacy Principles. The study’s data comprised of de-identified unit record administrative records. These were used under privileged arrangements set out in a study specific confidentiality deed. The data cannot be accessed by another party without relevant data custodian and human research ethics approvals.

## Abbreviations

95%CI: 95% confidence interval
BIC: Bayesian Information Criterion
CanDAD: Cancer Data and Aboriginal Disparities
ICD-O-3: International Classification of Diseases for Oncology
ISAAC: Integrated SA Activity Collection
MBS: Medicare Benefit Schedule
NSW: New South Wales
OR: odds ratio
PBS: Pharmaceutical Benefits Scheme
SA: South Australia
SACR: South Australian Cancer Registry
SEER: Surveillance, Epidemiology, and End Results Program
SHR: sub-hazard ratios
